# Assessment of the Utility of Whole Genome Sequencing of Measles Virus in the Characterisation of Outbreaks

**DOI:** 10.1371/journal.pone.0143081

**Published:** 2015-11-16

**Authors:** Ana Raquel Penedos, Richard Myers, Besma Hadef, Farah Aladin, Kevin E. Brown

**Affiliations:** Virus Reference Department, Public Health England, London, NW9 5EQ, United Kingdom; Centro Nacional de Microbiología - Instituto de Salud Carlos III, SPAIN

## Abstract

**Background:**

Measles is a highly infectious disease caused by measles virus (MeV). Despite the availability of a safe and cost-effective vaccine, measles is one of the world-leading causes of death in young children. Within Europe, there is a target for eliminating endemic measles in 2015, with molecular epidemiology required on 80% of cases for inclusion/exclusion of outbreak transmission chains. Currently, MeV is genotyped on the basis of a 450 nucleotide region of the nucleoprotein gene (N-450) and the hemagglutinin gene (H). However, this is not sufficiently informative for distinguishing endemic from imported MeV. We have developed an amplicon-based method for obtaining whole genome sequences (WGS) using NGS or Sanger methodologies from cell culture isolates or oral fluid specimens, and have sequenced over 60 samples, including 42 from the 2012 outbreak in the UK.

**Results:**

Overall, NGS coverage was over 90% for approximately 71% of the samples tested. Analysis of 32 WGS excluding 3’ and 5’ termini (WGS-t) obtained from the outbreak indicates that the single nucleotide difference found between the two major groups of N-450 sequences detected during the outbreak is most likely a result of stochastic viral mutation during endemic transmission rather than of multiple importation events: earlier strains appear to have evolved into two distinct strain clusters in 2013, one containing strains with both outbreak-associated N-450 sequences. Additionally, phylogenetic analysis of each genomic region of MeV for the strains in this study suggests that the most information is acquired from the non-coding region located between the matrix and fusion protein genes (M/F NCR) and the N-450 genotyping sequence, an observation supported by entropy analysis across genotypes.

**Conclusions:**

We suggest that both M/F NCR and WGS-t could be used to complement the information from classical epidemiology and N-450 sequencing to address specific questions in the context of measles elimination.

## Introduction

Infection with measles virus (MeV) causes measles, a highly contagious disease which, in the absence of vaccination, mainly affects children. Despite the availability of a safe and effective vaccine, measles is one of the leading causes of child death worldwide, particularly in poor nutrition and inefficient health care systems settings [1, 2]. All measles virus isolates characterised to date belong to a single serotype and both natural infection and vaccination produce lifelong immunity against the virus [3, 4]. The increase in vaccination rates has led to a dramatic reduction in measles deaths: in the period between 2000 and 2013 a 75% reduction in measles deaths was recorded (from 544,200 to 145,700 deaths/year), with an estimated 15.6 million deaths prevented in this period [5]. However, given the high infection rate of measles virus, outbreaks occur in populations where the vaccination coverage is lower than the 95% that confers herd immunity [6].

**Fig 1:**
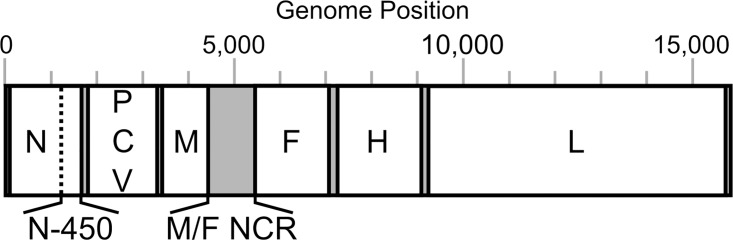
Schematic representation of the measles virus genome. The 15,894 nucleotides (nt) of the measles virus genome encode: nucleoprotein (N; 525 aa), phosphoprotein (P; 507 aa), matrix (M; 335 aa), fusion (F; 550 aa), hemagglutinin (H; 617 aa), large polymerase (L; 2,183 aa), C (299 aa) and V (186 aa) proteins. Coding regions of the genome (in white) are separated by non-coding regions (NCR; in grey). The longest NCR is that between the M and F genes: M/F NCR (1,012 nt). (aa used as abbreviation for amino acid)

The measles virus is a member of the *Morbilivirus* genus of the *Paramyxoviridae* family. It has a linear negative sense single-stranded RNA ((-)ssRNA) genome of 15,894 nucleotides (nt) which encodes six structural and two non-structural proteins (Fig 1) [7]. The structural proteins are the nucleoprotein (N), phosphoprotein (P), matrix (M), fusion (F), hemagglutinin (H) and large polymerase (L) proteins. The non-structural C and V proteins are both sub-products of the P gene: C results from an overlapping reading frame and V from an edited transcript. Each coding region is preceded and followed by untranslated regions, of which the longest (1,012 nt) is the non-coding region between the genes for the M and F proteins (M/F NCR).

Measles virus genotyping, in association with epidemiology data, is recommended by the World Health Organization (WHO) in 80% of chains of transmission for measles surveillance and outbreak control [8, 9]. Genotyping is based on a 450 nucleotide window located in the nucleoprotein gene (N-450) (Fig 1) and the H gene is used in conjunction with N-450 for the identification of new genotypes and as an auxiliary in outbreak characterisation [10]. Sequence data collected across all laboratories reporting to WHO is submitted to the measles nucleotide surveillance database (MeaNS; http://www.who-measles.org) [11], where it can be analysed. The results of these analyses, in conjunction with epidemiological data, play an essential role in the identification of transmission pathways and patterns.

The WHO has set the goal for measles elimination (interruption of measles transmission for over one year) in the European region for 2015 [12]. Although 24 genotypes of measles virus have been identified (A, B1-3, C1-2, D1-11, E, F, G1-3, H1-2), only a fraction of these is currently circulating [10, 11, 13]. As the elimination of measles draws closer in Europe, the diversity in circulating measles virus is decreasing and the information provided by the N-450 genotyping window is increasingly proving insufficient in the description of outbreaks as often little or no variation in the sequence is detected for several months or years (e.g.: MVs/Enfield.GBR/14.07[D4], 2007–2012; MVs/Manchester.GBR/10.09[D4], 2009–2014; MVi/Harare.ZWE/38.09[B3], 2009 to present) [13]. To demonstrate interruption of measles circulation, countries must be able to distinguish between endemic transmission and importation events. The widening of the sequencing window for measles virus may then be necessary to verify measles elimination.

In the United Kingdom (UK), endemic transmission of MeV was interrupted between 1995 and 2001 [14]. However, following a decrease in vaccination coverage, the number of confirmed measles cases has increased and several outbreaks have since been described in susceptible populations [15–19]. In England, measles surveillance is carried out at the Virus Reference Department of Public Health England (PHE). For 90% of suspected cases, a patient oral fluid is submitted, allowing IgM and IgG detection as well as PCR testing and genotyping [20, 21]. Currently, genotypes B3 and D8 of MeV account for the majority of cases of measles in the UK (MeaNS; http://www.who-measles.org) [11].

In this study, we use a MeV-specific amplicon-based method for whole genome sequencing directly from clinical specimens by a combination of Sanger and next generation sequencing (NGS) technologies to assess the utility of a wider sequencing window in the characterisation of measles outbreaks. Samples from genotypes B3 and D8 with similar N-450 sequences were selected and phylogenetic analysis of several regions of the genome was carried out to identify regions of higher variability.

## Materials and Methods

### Measles virus specimens and isolates

All specimens used were sent to the Immunisation and Diagnosis Unit at Public Health England in the course of routine surveillance and diagnosis work. 62 of the 73 samples selected (S1 Table) were oral fluids previously confirmed as measles-positive by PCR. The remaining 11 samples were isolated in the Vero/hSLAM cell line as described earlier [21].

In order to address the significance of differences observed in N-450 sequences, samples were selected to represent genotypes D8, D4 and B3 (S1 Table). D8 strains (n = 51) were selected mainly from the samples collected in the early, mid and late stages of a large measles outbreak that occurred in England and Wales in 2012–13 (n = 50), representing N-450 sequences observed at different stages of the outbreak. When available, three strains were selected to represent a cluster at each time point.

The D4 strains (n = 11) selected date back to a measles outbreak in Manchester in 2011 and share identical N-450 sequence. Finally, strains of the B3 genotype (n = 11) were selected from samples collected in late 2013 –early 2014 and represent a cluster of measles cases in London.

### RNA extraction, PCR and Sequencing

Viral RNA was extracted using the QIAamp Viral RNA Mini kit (Qiagen^®^, 52904). The kit protocol was followed with the following changes: volume of AVL with carrier solution used, 400 μl; volume of sample to extract, 100 μl; volume of ethanol added, 400 μl; volume of AVE buffer for elution, 2x 40 μl. Following extraction, RNA was stored at -80°C or immediately used as the template for a one-step block-based PCR.

Viral titre was estimated using measles H gene real-time RT-PCR (measles qRT-PCR) as described before [21]. The OneStep RT-PCR kit (Qiagen^®^, 210212) was used for reverse transcription and amplification of the template. Measles-specific primers designed to amplify measles genotypes B3, D4 and D8 (S2 Table) were used to amplify the genome in 20 overlapping amplicons of approximately 1 kb each. The cycling conditions were: 30 min at 50°C, 15 min at 95°C, 40 cycles of 80 s at 94°C, 90 s at 55°C and 2 min at 72°C, and a final elongation of 10 min at 72°C. To obtain the seventh amplicon, a second PCR round was carried out using the Taq PCR Master Mix kit (Qiagen^®^, 201443). For the second round PCR, the cycling conditions were: 3 min at 94°C, 40 cycles of 80 s at 94°C, 90 s at 55°C and 2 min at 72°C, and a final elongation of 10 min at 72°C.

The PCR products were checked by standard DNA electrophoresis techniques and purified using the Agencourt AMPure XP PCR purification kit (Beckman Coulter^®^, A63880) according to the kit’s instructions. Sample concentrations were measured and adjusted for Sanger or Illumina^®^ MiSeq next generation sequencing (NGS). All amplicons for each sample were pooled for NGS, concentration was determined using Quant-iT DNA High Sensitivity Assay Kit (Life Technologies^®^, Q33120) and library preparation was done using the Nextera XT DNA Sample Preparation Kit (Illumina^®^, FC-131-1024). Sanger sequencing was used to complete NGS sequences, particularly across the non-coding region between the matrix and fusion protein genes (M/F NCR; Fig 1).

### NGS data processing

Illumina^®^ FASTQ files were reference-mapped to measles virus (MeV) genotype-specific consensus genomes using BWA (version 0.7.5) [22]. Genotype-specific consensus sequences were derived from MeV genome sequences deposited in GenBank. SAM files produced by reference mapping were converted into BAM files using Samtools (version 1.1.2) [23]. BAM files were processed using an in-house C++ program (Quasibam) to derive a consensus sequence with mixtures greater than 20% coded as IUPAC ambiguities and a text file containing a table of nucleotide frequency, depth and quality metrics for each nucleotide position in the mapping process. Ambiguities introduced into the consensus sequence by the PCR amplification process were removed by comparing primer sequences with the frequency of nucleotides mapped in primer positions. Where a primer mismatch was likely to have introduced a minority variant into the consensus sequences, these variants were removed from further analysis.

### Phylogenetic analysis and epidemiological clustering

Phylogenetic analysis was carried out only on strains for which the whole genome sequence between the first and last coding nucleotides (i.e., excluding 3’ and 5’ genome termini; WGS-t) was obtained. The length of WGS-t sequences is 15,678 nucleotides (nt) for the B3 and D8 MeV genotypes used in this study. The sequences were deposited in GenBank and accession numbers are provided in S1 Table. Maximum likelihood trees for the WGS-t, N, N-450, P, M, M/F NCR, F, H and L regions of the measles genome (Fig 1) were obtained with RAxML using the generalised time-reversible (GTR) model of nucleotide substitution and 1000 bootstraps. Dated trees were also generated for the D8 WGS-t and M/F NCR sequence alignments using BEAST (v1.8.2). Trees were generated using the GTR nucleotide substitution model, a lognormal relaxed molecular clock and a constant coalescence model. 20 million chains were run for the WGS-t sequences and 10 million for the M/F NCR alignment. A maximum clade credibility tree was generated for each analysis.

Phylogenetic trees were coloured according to classical epidemiology clusters, where known contacts or individuals attending the same events or institutions in the relevant transmission period are grouped together as described in a previous study [14].

### Entropy and substitution rate analyses

Entropy plots for the D8 outbreak sequences and all whole genome sequences available for non-A genotypes in GenBank were obtained using an in-house script that calculates the entropy at each nucleotide position in an alignment, averaged over a window of 100 bases (50 base steps).

Substitution rates were calculated using BEAST (see above) and by determining the number of differences between each unique sequence and a BEAST analysis-derived ancestor sequence. The number of differences found was plotted against the earliest date at which each sequence was detected and used to estimate a substitution rate. The date for each sample was extracted from the WHO strain name.

## Results and Discussion

### Majority of sequence can be obtained from clinical specimens with wide range of viral titres

**Fig 2:**
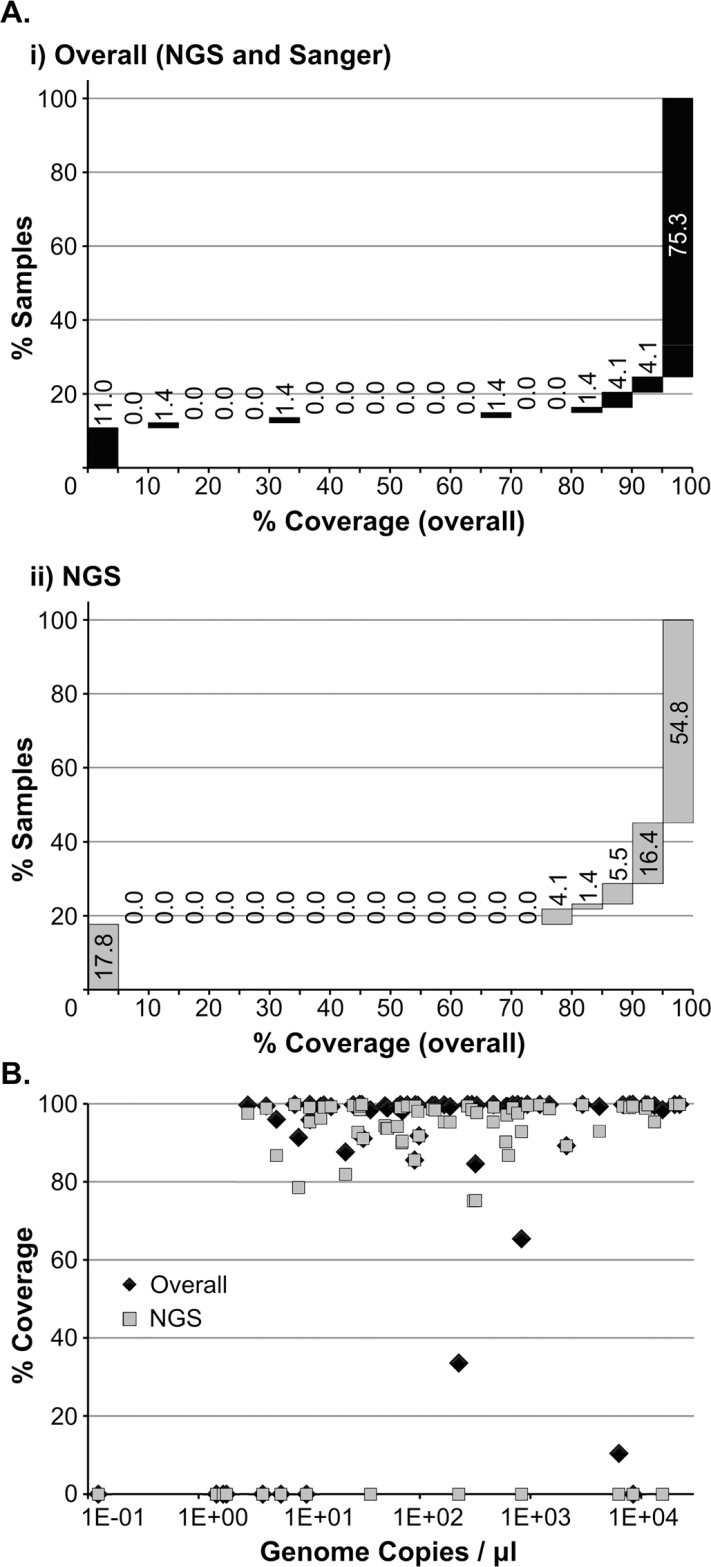
Sequencing outcomes. (A) Percentage of samples for which the coverage obtained falls within each 5% coverage window following sequencing by NGS and Sanger (i) or NGS only (ii). Bars exclude lower category value and include higher category value (except first bar, which includes 0). (B) The fraction of the genome obtained (% coverage) by a combination of NGS and Sanger (black diamonds) or NGS only (grey squares) is plotted against sample viral titre (genome copies / μl).

Patient specimens and cell culture isolates representing measles virus (MeV) genotypes D8, B3 and D4 (see methods) were submitted to RT-PCR and sequenced using NGS. Sanger sequencing was used to close gaps in sequences. The percentage of the measles genome covered by the combination of NGS and Sanger technologies was over 90% for 58 of the samples selected (79.5%) (Panel Ai in Fig 2). The fraction of samples for which this coverage is obtained solely by NGS is reduced to 71.2% (Panel Aii in Fig 2), mainly due to the difficulty in obtaining the non-coding region situated between the genes coding for the matrix and fusion proteins (M/F NCR). The PCR multiplexing carried out prior to NGS also impaired the ability to adjust amplicon concentrations for an optimal coverage by this method and reduced the sensitivity of the PCR. In general, failure in obtaining good quality sequence data was associated with weaker or unspecific amplification. However, for the M/F NCR, strong, specific amplification was not always conductive to good quality NGS sequence data. The reason for this is not clear, but may be associated with a complex secondary structure in this genomic region as it shows a higher GC content than the remainder of the genome (63% and 47%, respectively).

No correlation was found between sample titre and the fraction of the genome covered (Panel B in Fig 2): of the 15 samples for which overall genome coverage was under 90%, 7 had a titre lower than 10 genome copies/μl and the remaining 8 had viral titres between 21.1 and 8,414 genome copies/μl. The viral titres for samples for which over 90% of the genome sequence was obtained ranged from 2.8 to 22,261 genome copies/μl, corresponding to measles real-time RT-PCR cycle threshold values between 32 and 19. Thus, our MeV-specific amplification method allows for the sequencing of patient specimens with a wide range of viral loads. The failure to successfully amplify samples across this range is likely to be associated with inhibitors present in the sample. This is, to our knowledge, the first study where a method which allows for sequencing MeV directly from low titre patient specimens is used.

### M/F NCR and N-450 afford the highest phylogenetic resolution

Phylogenetic analysis was carried out for all the strains for which the whole genome sequence excluding the 3’ and 5’ termini (WGS-t, 15,678 nt) was available. These included 32 measles strains of genotype D8 and 11 of B3. All WGS-t were analysed for each gene individually, for the M/F NCR and as a whole. Phylogenetic analysis is not shown for D4 strains given that only 5 WGS-t sequences were obtained for this genotype.

**Fig 3:**
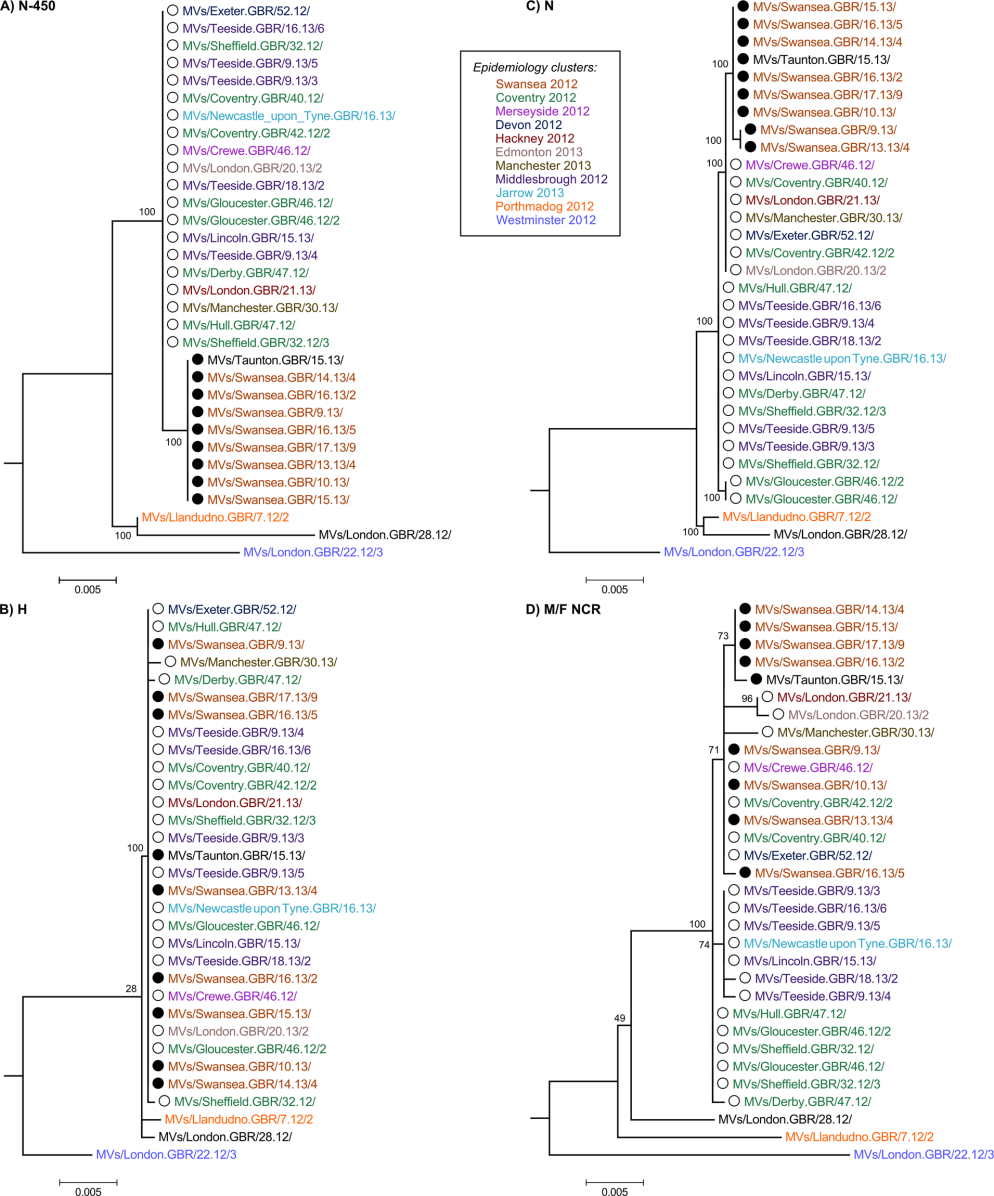
Phylogenetic analysis of the N-450, H, N and M/F NCR sequences of D8 outbreak strains. The 32 D8 strains for which the whole genome sequence excluding the 3’ and 5’ termini (WGS-t) was available were analysed using RAxML. Phylogenetic trees were obtained for the N-450 (A), H (B), N (C) and M/F NCR (D) sequences. Strains are coloured according to the epidemiology clusters in which they were grouped.

**Fig 4:**
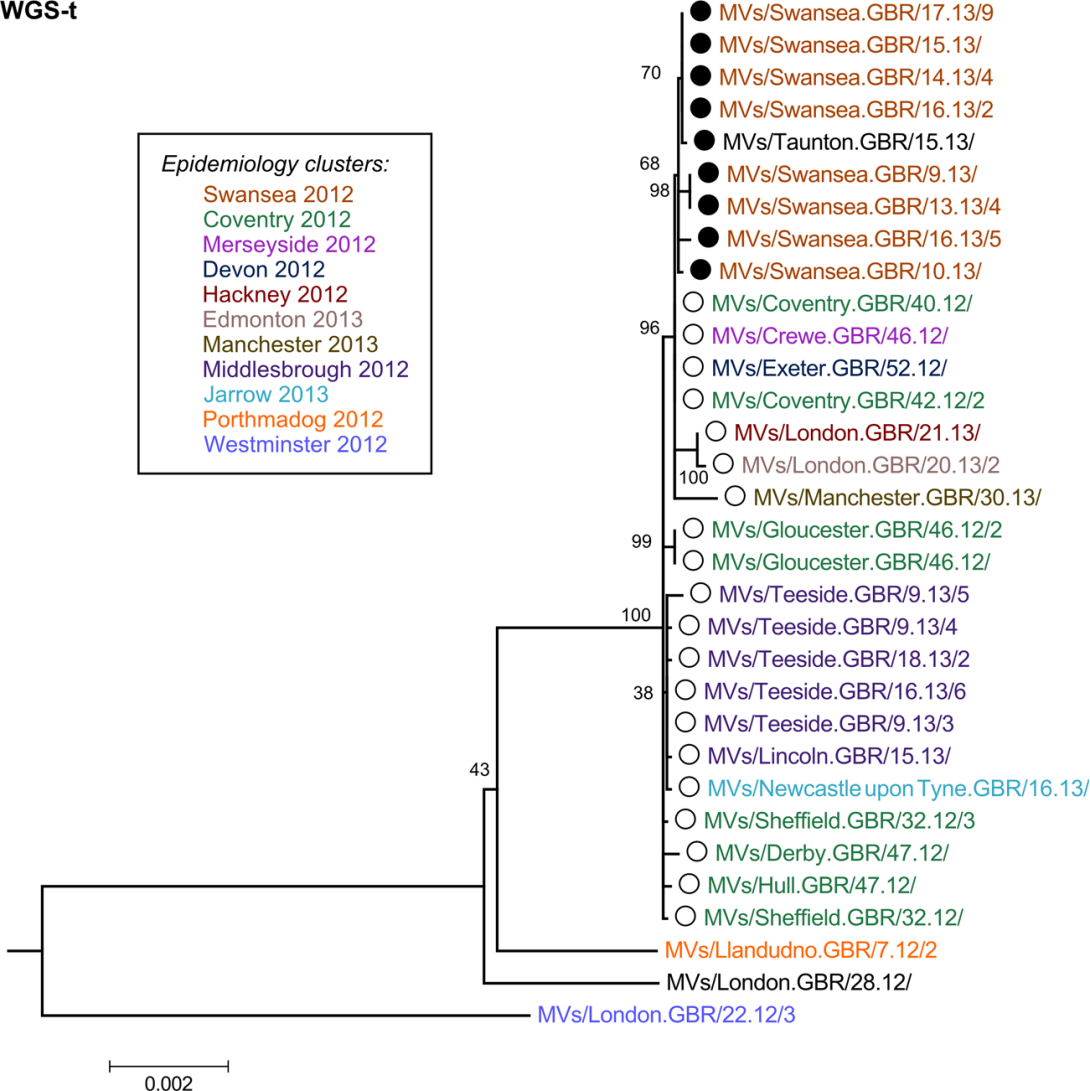
Phylogenetic analysis of the WGS-t sequence of D8 outbreak strains. Phylogenetic analysis was carried out as described for Fig 3. A smaller scale is used for the WGS-t phylogenetic tree given the lower rate of changes/site.

The majority of the strains collected throughout the D8 outbreak in 2012–13 have identical N-450 sequence to the WHO named strains MVs/Taunton.GBR/27.12/ (henceforth referred as Taunton; marked with ○ in the phylogenetic and Bayesian trees) and MVs/Swansea.GBR/4.13/ (henceforth referred as Swansea; marked with ● in the phylogenetic and Bayesian trees) (n = 38/50) (Panel A in Fig 3 and S1 Table), which have sequences that differ in a single nucleotide at position 1322 (guanine in Taunton and adenine in Swansea strains). The analysis of the H gene, used in conjunction with N-450 for the genotyping of MeV [10], does not provide significant further separation of the strains analysed (Panel B in Fig 3), with the majority of strains belonging to the Taunton and Swansea clusters showing identical H sequences. The phylogenetic tree obtained from the whole of the N gene (Panel C in Fig 3) appears to produce better resolution of the strains analysed compared to that obtained from the H gene. The region of the genome that originates the most detailed phylogeny relatively to the length of sequence analysed is the non-coding region located between the M and F genes (M/F NCR) (Fig 1 and Panel D in Fig 3). In fact, the tree resolution obtained for the M/F NCR (1,012 bp) is akin to that resulting from the analysis of the WGS-t (15,678 bp) (Fig 4). Comparatively, the phylogenetic trees obtained from the analyses of the P, M and F genes yield very low distinction between strains (Panels A-C in S1 Fig). Analysis of the L gene allows better phylogenetic resolution (Panel D in S1 Fig), which would be expected given the high fraction of the measles genome represented by this gene (>40%; Fig 1).

Phylogenetic analysis of the strains from genotype B3 suggests that, in this context, the H gene may be more informative than the whole of the N gene (Panels B and C in S2 Fig), an observation supported by a previous study [24]. The M/F NCR sequence provides, as in D8 strains, good phylogenetic tree resolution (Panel D in S2 Fig). For the B3 sequences available, phylogenetic analyses of both P and M genes (Panels A and B in S3 Fig) afford more detail than that obtained in strains of genotype D8 (Panels A and B in S1 Fig).

It is not clear why there would be differences in variability in genomic regions (e.g., H, P and M genes) across genotypes. It has been shown that the F, H and L genes are subject to strong constrains in terms of mutation tolerance [25]. A previous study of an outbreak of MeV genotype B3 in Spain [24] has found the sequence of the H gene to be highly variable and informative. One may speculate that the H gene in MeV genotype B3 could be more variable than in other genotypes, but further WGS data collected over a longer period of time and a wider region would be required to address this question.

### 2012–13 D8 outbreak in England and Wales most likely resulted from endemic transmission

The analyses of the strains associated with the D8 outbreak in England and Wales in 2012–13 suggests that two phylogenetic branches develop from the samples collected early in the outbreak (2012; Panel D in Figs 3 and 4). The first of these branches contains samples belonging exclusively to the N-450 Taunton cluster. The second branch contains strains from both Taunton and Swansea N-450 clusters. No strains with identical N-450 sequence to the Swansea strains were reported before the first sample was collected in the UK. Three weeks later, a small cluster of cases was reported in Canada with the same N-450 sequence (GenBank accession numbers KC759182 and KF672749). These observations suggest that the strains in the Swansea cluster have resulted from genetic drift (the process by which presumed neutral mutations in the measles virus genome are fixed within a host’s viral quasispecies by chance rather than by positive selection) of the Taunton strains in the second branch.

**Fig 5:**
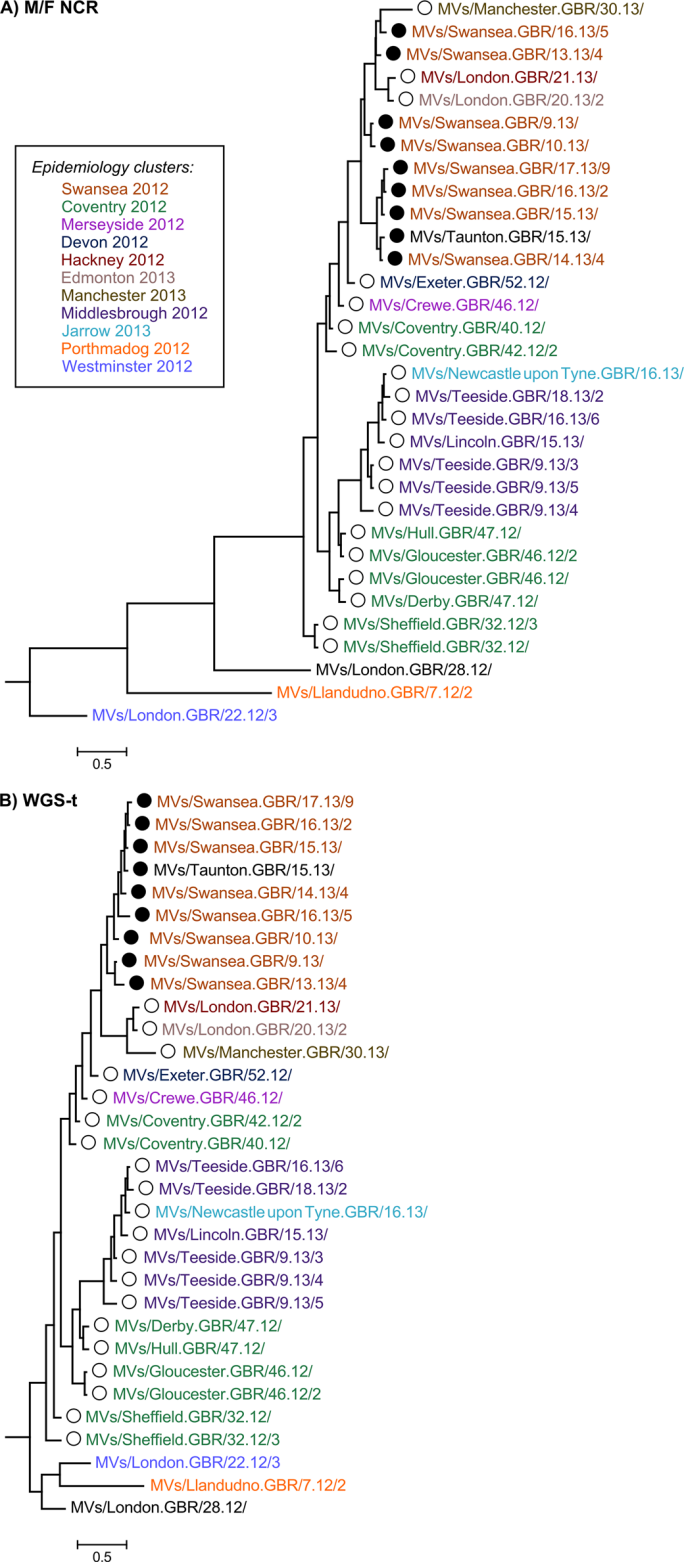
BEAST analysis of D8 strains. Analysis of the M/F NCR (A) and WGS-t (B) sequences of the D8 strains in this study was carried out using the week and year of sample collection as a measurement of sample time.

The fact that cases occurred later in the outbreak (2013) appear to have ancestor strains earlier in the outbreak (2012) suggests that they are likely to result from a chain of measles transmission rather than from separate importation events. This interpretation is supported by the BEAST analysis of the M/F NCR and WGS-t, where two Sheffield strains collected on week 32 of 2012 appear to share a common ancestor with all the remaining strains associated with the outbreak (Fig 5).

The analysis of an extended sequencing window, including either the M/F NCR or the WGS-t, complements the information provided by both the N-450 region and the epidemiological data available. The comparison of the clusters obtained by phylogenetic analysis of the first two regions with those obtained from epidemiological analysis of cases (colour-coded in Figs 3 and 4 and S1 Fig) shows that the majority of the phylogenetic clusters match epidemiological groupings, with Swansea 2012 and Middlesbrough 2012 strains grouped together by both approaches. However, the phylogenetic analysis indicates that some of the strains that were thought to be epidemiologically linked may indeed be further apart in genetic terms as evidenced by the distribution of Coventry 2012 strains between two distinct phylogenetic clusters (Panel D in Figs 3 and 4).

### M/F NCR and N-450 are the most variable genomic regions across genotypes

**Fig 6:**
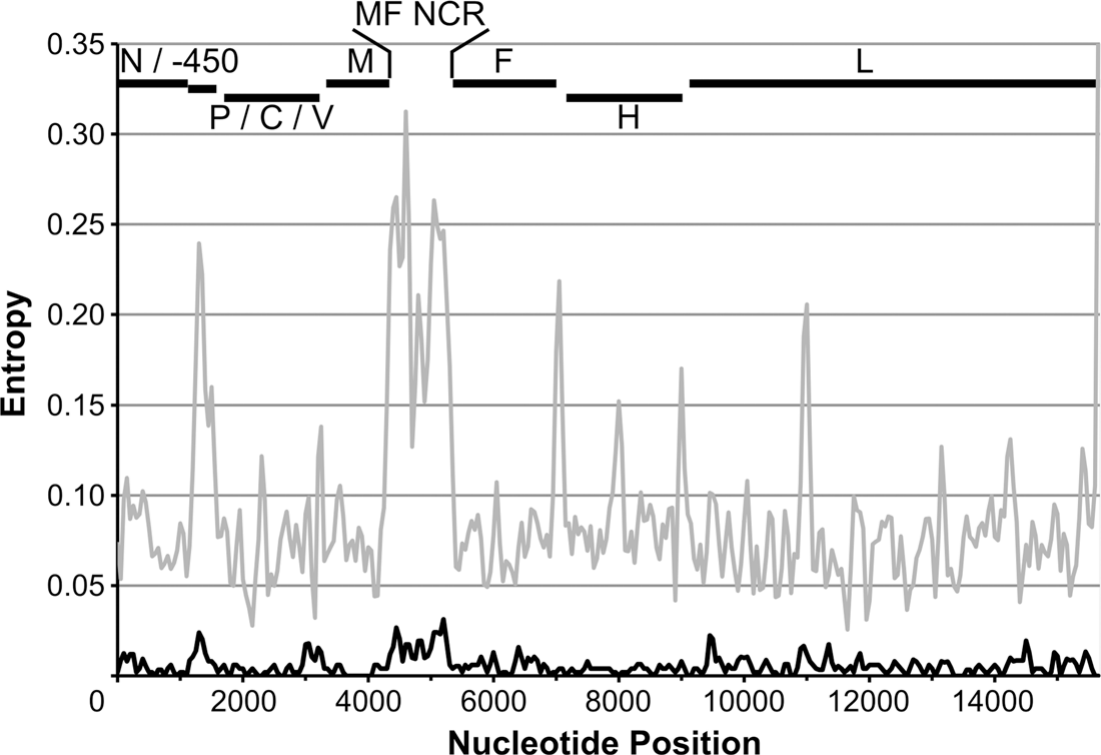
Sequence variability in the measles virus genome. Shandon entropy plot representing nucleotide variability at each position of the measles genome (average over a sliding window of 100 nucleotides with a step of 50 bases). Analysis was carried out for D8 (black line) and all available measles genome sequences in GenBank for measles genome sequences from non-A genotype (grey line).

The variability of nucleotides observed at each position of the measles virus genome was assessed by analysis of sequence entropy at each genome position (Fig 6). For the D8 samples analysed, the regions where higher variability is observed are located at the N-450, in the region of the P gene encoding the carboxyl terminus of the P protein, at the M/F NCR, along the F gene and in several regions of the L gene (black line in Fig 6). Comparison of this plot to that obtained for all complete genome sequences from non-A measles genotypes (given that A genotype sequences are mainly vaccine strains) available in GenBank (grey line in Fig 6) suggests that N-450 and M/F NCR are the most variable regions in the genome across all measles genotypes, in agreement with the observations from phylogenetic analyses (Figs 3 and 4 and S1–S3 Figs) and previous studies [26–30].

BEAST was used to obtain an estimate of the substitution rate observed at the M/F NCR and WGS-t during the D8 outbreak. Given the reduced number of N-450 samples associated with the outbreak studied here, the substitution rate for this region was obtained for all UK sequences available in MeaNS (http://www.who-measles.org) [11] for genotypes D1-11. As a second approach, the number of differences between each unique sequence and an ancestor obtained through the BEAST analysis was plotted against sample date to estimate a substitution rate (Table 1). Both approaches suggest that the substitution rate (substitutions/site/year) is highest for the M/F NCR: approximately 3-fold higher than that for the N-450 by both estimates. The ratio between M/F NCR and WGS-t substitution rates is 5.6 by the number of differences approach and 1.7 by BEAST analysis.

**Table 1:** Substitution rates for measles virus N-450, M/F NCR and WGS-t genomic regions.

Sequence set	Region	substitutions/site/year	
		BEAST	# differences
D8 outbreak	M/F NCR	6.5 x 10^−3^	2.8 x 10^−3^
D8 outbreak	WGS-t	3.9 x 10^−3^	5.0 x 10^−4^
D UK MeaNS	N-450	1.8 x 10^−3^	9.6 x 10^−4^

## Conclusions

Establishing whether measles cases result from several importations of the virus rather than from a continuous chain of transmission within the country provides important information to more timely allocation of resources. While importations of MeV result in a reduced number of cases, no action is likely to be necessary in augmenting the immunity of the population. However, if one imported case then triggers an outbreak, this suggests sub-optimal population immunity. In this instance, a susceptible group may then be identified and potentially vaccinated to prevent further spread of the virus. Study of cases by the classical epidemiology approach provides important contact and travel information, but this may be insufficient to arrive to a conclusion as to the provenance of the virus. Additional information can be obtained from the sequencing of the N-450 region, which provides crucial information on global measles circulation and assists in the characterisation and tackling of countless outbreaks worldwide, for example by enabling clinicians and epidemiologists to identify and distinguish between cases resulting from simultaneous outbreaks of different MeV genotypes. However, in view of the decrease in the diversity of the circulating MeV as several regions of the WHO endeavour to eliminate measles, it is likely that the diversity of the N-450 sequences will also decrease [30, 31]. In this context, additional tools for molecular characterisation would be required.

In this study, we used a new MeV-specific PCR enrichment method for NGS and Sanger which permits sequencing of the whole genome directly from patient oral fluid specimens with viral titres as low as 2.8 genome copies/μl, thus eliminating the need to culture viral isolates and, consequently, culture-associated mutation biases [32]. In order to assess the utility of a wider sequencing window for MeV in the context of low sequence diversity, the samples selected for analyses shared identical N-450 sequences. The majority of samples represent MeV genotype D8 and were collected during the large outbreak in England and Wales in 2012–13. Phylogenetic analysis of 32 samples suggests that endemic transmission and not multiple importation events explain the outbreak (Figs 3–5). This observation illustrates the potential advantage of using a wider sequencing window in answering specific epidemiological questions in the context of reduced MeV diversity.

The analysis of various regions of the MeV genome in genotype D8 and B3 strains indicates that, as expected, the most information can be derived from the WGS-t. However, the phylogenetic resolution afforded by this region is closely followed by that obtained with the M/F NCR. The nucleotide variability observed at each genome position and substitution rates estimated for each of these sequences support the notion that the M/F NCR and the N-450 are the most variable regions across all genotypes (Fig 6) [26–30]. Interestingly, we also find that the variability of specific regions of the genome differs between MeV D8 and B3 genotypes, with the whole of the N gene being more variable in the first and the H gene in the second, for example. The study of a wider range of strains collected worldwide at various time points would be necessary to further address potential reasons and consequences of these differences across genotypes.

While WGS would provide the most information for distinction between outbreak strains, current limitations of NGS technology mean that samples must be enriched prior to library preparation. PCR has been the prevalent choice for enrichment, but covering a full genome with amplicons of a size allowing for good sensitivity (20 amplicons of approximately 1kb in the present case) can be costly and time-consuming. WGS using the Sanger methodology would be at least as resource-intensive as the NGS alternative given the need for a large number of sequencing reactions for each sample (40 in this case). Additionally, data analysis is not straightforward, in particular in the case of NGS [33–35]. WGS-t sequencing may thus be time- and cost-inefficient and most likely only feasible in specific circumstances in well-resourced countries which have already reached or are approaching measles elimination.

A potential alternative for countries where N-450 sequencing is already carried out routinely and where more detail is needed in a low MeV diversity scenario might be the use of a smaller highly-variable region of the genome in addition to the N-450. Our results suggest that the non-coding region located between the M and F genes appears to be the one from which most information could be gathered, as the phylogenetic trees produced based on its 1,012 nt sequence show a resolution comparable to that of those based on the 15,678 nt of the WGS-t. Given that a nested PCR followed by Sanger sequencing is effective in obtaining the M/F NCR sequence, this might be the most time- and cost-effective approach for obtaining additional information when this is required.

Although the WHO has recommended that genotyping is carried out in 80% of the chains of transmission, resource-limitations mean that this is rarely achieved. The priority should be to first implement routine N-450 sequencing. This region, being shorter and easier to obtain than the M/F NCR or the WGS-t is one of the most variable regions in the MeV genome and should provide sufficient information where measles is still endemic. Neither the M/F NCR nor the WGS-t will ever replace the efficient collection of epidemiological and genotyping data. Only countries where genotyping is already carried out in a routine basis and where measles has been eliminated should consider the use of a wider sequencing window for MeV. Even in this context, WGS may prove too resource-intensive to be carried out routinely until improvements are made to NGS processes that render them cheaper and more feasible. The sequencing of the M/F NCR is, for the moment, more attainable and could be used to address specific epidemiological questions on an infrequent basis. However, further investigation into the number of differences that must be found in this region to eliminate direct transmission is necessary to evaluate if the estimated substitution rate would make it a viable auxiliary tool in outbreak characterisation. Until then, both WGS-t and M/F NCR sequencing will be useful research tools that could be employed in the better understanding of MeV evolution.

## Supporting Information

S1 Fig Phylogenetic analysis of the P, M, F and L sequences of D8 strains. The 32 D8 strains for which the whole genome sequence excluding the 3’ and 5’ termini (WGS-t) was available were analysed using RAxML. Phylogenetic trees were obtained for the P (A), M (B), F (C) and L (D) sequences.
(TIF)

S2 Fig Phylogenetic analysis of the N-450, H, N, M/F NCR and WGS-t sequences of B3 strains. The 11 B3 strains for which the whole genome sequence excluding the 3’ and 5’ termini (WGS-t) was available were analysed using RAxML. Phylogenetic trees were obtained for the N-450 (A), H (B), N (C), M/F NCR (D) and WGS-t (E) sequences.
(TIF)

S3 Fig Phylogenetic analysis of the P, M, F and L sequences of B3 strains. Phylogenetic analysis was carried out as described for S2 Fig. Phylogenetic trees were obtained for the P (A), M (B), F (C) and L (D) sequences.
(TIF)

S1 Table Samples selected for this study. enBank accession numbers are provided for samples for which the whole genome sequence excluding the 3’ and 5’ termini (WGS-t) is available. When a WHO named strain with identical N-450 sequence has been identified, this is indicated.
(PDF)

S2 Table Primers used for PCR enrichment. (PDF)

## Abbreviations

F: fusion protein
H: hemagglutinin protein
L: large polymerase
M: matrix protein
MeaNS: measles nucleotide surveillance database
MeV: measles virus
M/F NCR: non-coding region located between the matrix and fusion protein genes
N: nucleoprotein
N-450: 450 nucleotide region of the nucleoprotein gene used for measles virus genotyping
NCR: non-coding region
NGS: Next Generation Sequencing
P: phosphoprotein
WGS-t: whole genome sequence excluding 3’ and 5’ termini
