# Supplementary figures and images for: Assessment of the Utility of Whole Genome Sequencing of Measles Virus in the Characterisation of Outbreaks
Source: PLoS One. 2015 Nov 16;10(11):e0143081. doi: 10.1371/journal.pone.0143081 (PMC4646484; doi:10.1371/journal.pone.0143081)

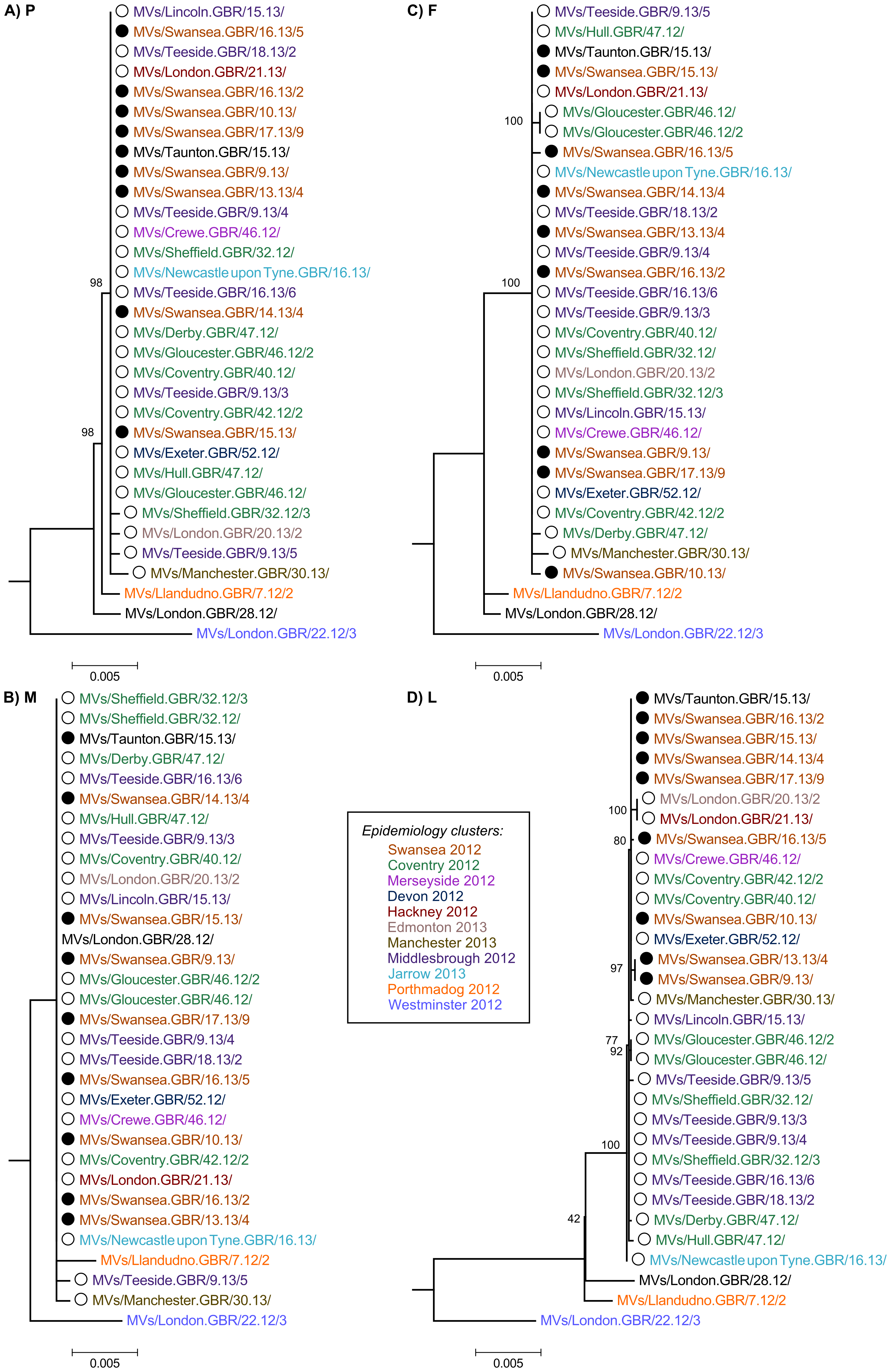

Supplement: S1 Fig — The 32 D8 strains for which the whole genome sequence excluding the 3’ and 5’ termini (WGS-t) was available were analysed using RAxML. Phylogenetic trees were obtained for the P (A), M (B), F (C) and L (D) sequences. (TIF) [file pone.0143081.s001.tif]

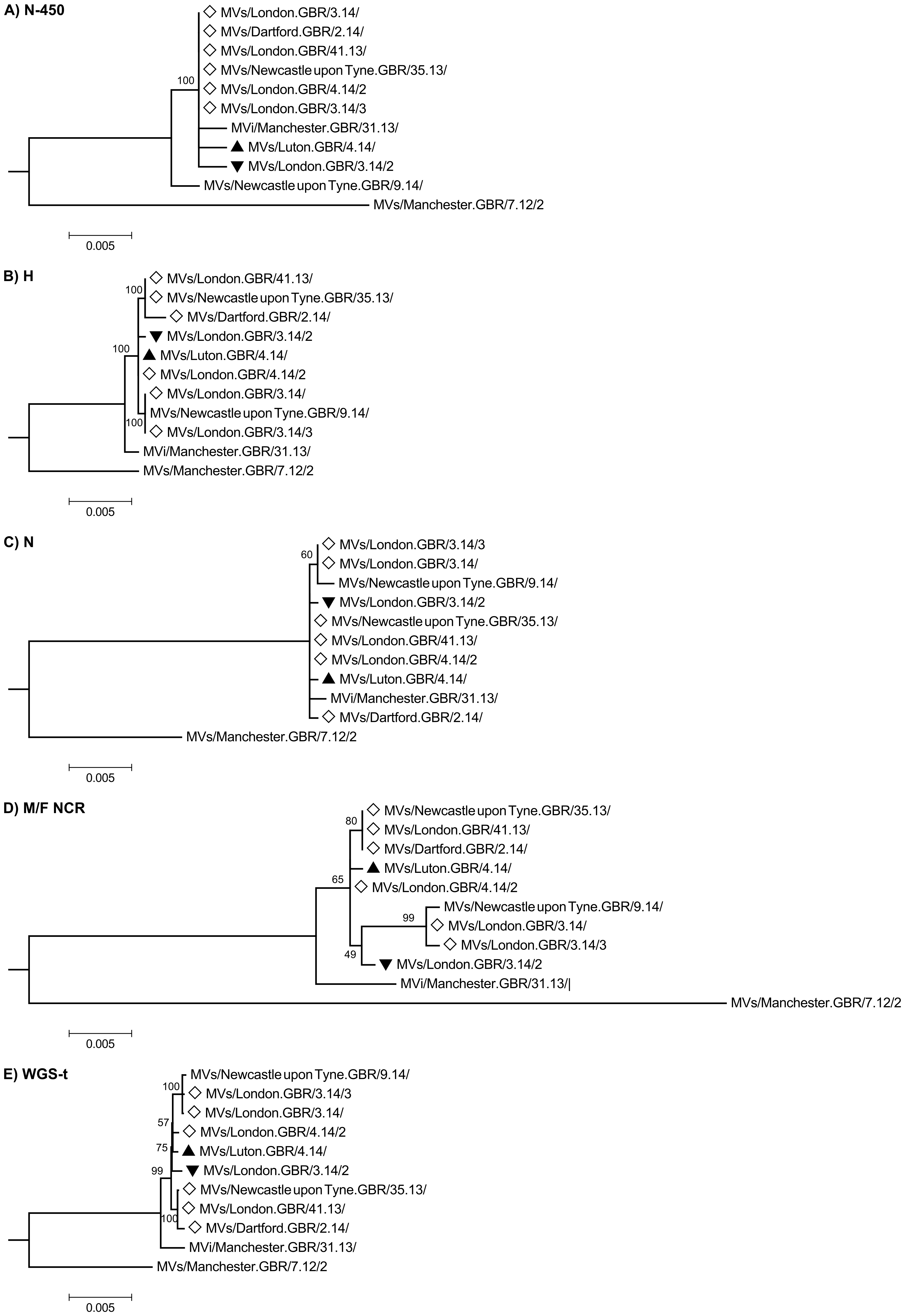

Supplement: S2 Fig — The 11 B3 strains for which the whole genome sequence excluding the 3’ and 5’ termini (WGS-t) was available were analysed using RAxML. Phylogenetic trees were obtained for the N-450 (A), H (B), N (C), M/F NCR (D) and WGS-t (E) sequences. (TIF) [file pone.0143081.s002.tif]

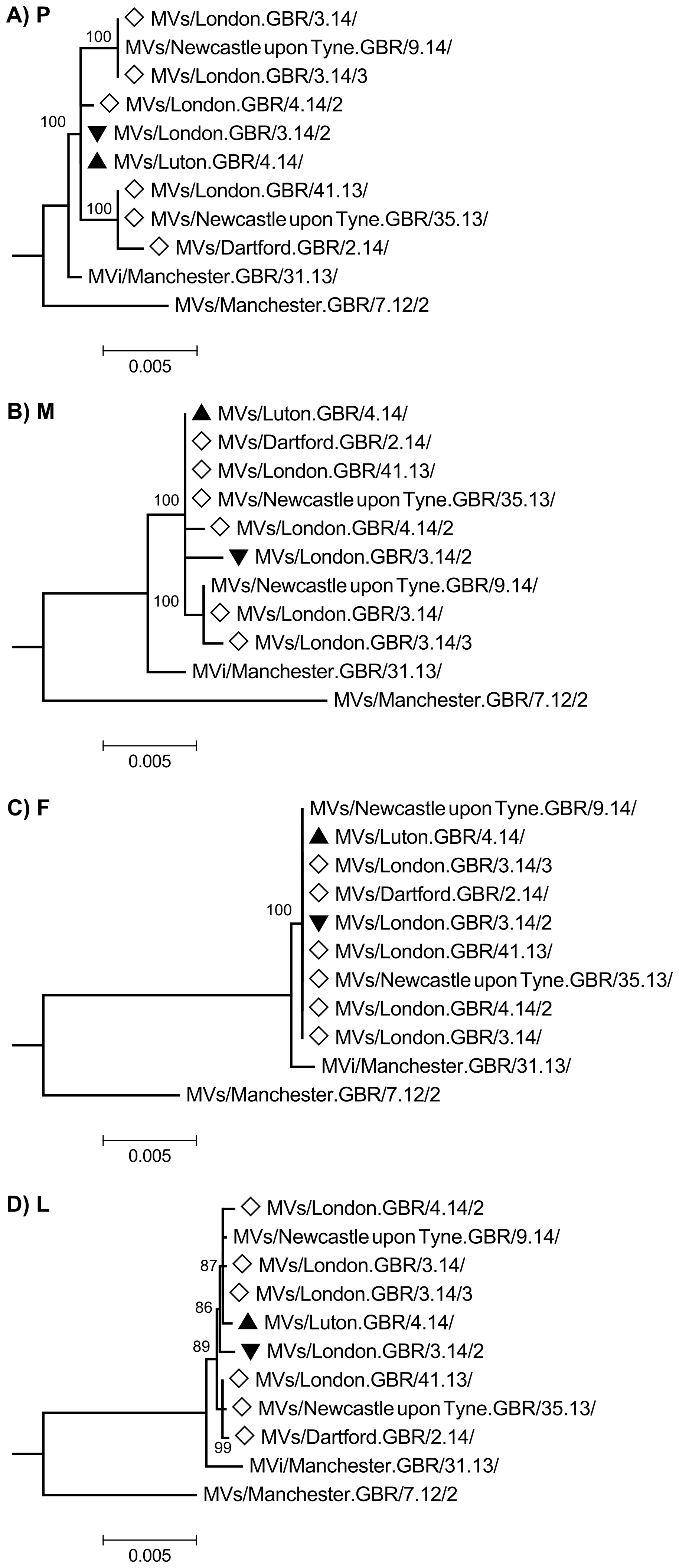

Supplement: S3 Fig — Phylogenetic analysis was carried out as described for S2 Fig. Phylogenetic trees were obtained for the P (A), M (B), F (C) and L (D) sequences. (TIF) [file pone.0143081.s003.tif]
