# Supplementary material for: Assessment of the Utility of Whole Genome Sequencing of Measles Virus in the Characterisation of Outbreaks
Source: PLoS One. 2015 Nov 16;10(11):e0143081. doi: 10.1371/journal.pone.0143081 (PMC4646484; doi:10.1371/journal.pone.0143081)
Supplement: S1 Table — enBank accession numbers are provided for samples for which the whole genome sequence excluding the 3’ and 5’ termini (WGS-t) is available. When a WHO named strain with identical N-450 sequence has been identified, this is indicated. (PDF) [file pone.0143081.s004.pdf]

S1 Table. Samples selected for this study.

GenBank accession numbers are provided for samples for which the whole genome sequence excluding the 3' and 5' termini (WGS-t) is available. When a WHO named strain with identical N-450 sequence has been identified, this is indicated.

Genotype	WHOname	Material	WHO named strain	GenBank
B3	MVs/Manchester.GBR/7.12/2	Cell culture	MVs/Liverpool.GBR/5.12/	KT732214
B3	MVi/Manchester.GBR/31.13/	Cell culture	n/d	KT732215
B3	MVs/Newcastle upon Tyne.GBR/35.13/	Oral fluid	MVi/Harare.ZWE/38.09/	KT732216
B3	MVs/London.GBR/41.13/	Cell culture	MVi/Harare.ZWE/38.09/	KT732217
B3	MVs/Dartford.GBR/2.14/	Oral fluid	MVi/Harare.ZWE/38.09/	KT732218
B3	MVs/London.GBR/3.14/	Oral fluid	MVi/Harare.ZWE/38.09/	KT732219
B3	MVs/London.GBR/3.14/2	Oral fluid	n/d	KT732220
B3	MVs/London.GBR/3.14/3	Oral fluid	MVi/Harare.ZWE/38.09/	KT732221
B3	MVs/Luton.GBR/4.14/	Oral fluid	n/d	KT732222
B3	MVs/London.GBR/4.14/2	Oral fluid	MVi/Harare.ZWE/38.09/	KT732223
B3	MVs/Newcastle upon Tyne.GBR/9.14/	Oral fluid	n/d	KT732224
D4	MVs/Leeds.GBR/9.11/2	Oral fluid	MVs/Manchester.GBR/10.09/	n/a
D4	MVs/London.GBR/17.11/4	Oral fluid	MVs/Manchester.GBR/10.09/	n/a
D4	MVs/London.GBR/19.11/5	Oral fluid	MVs/Manchester.GBR/10.09/	KT732225
D4	MVs/London.GBR/20.11/2	Oral fluid	MVs/Manchester.GBR/10.09/	KT732226
D4	MVs/Brighton.GBR/41.11/2	Cell culture	MVs/Manchester.GBR/10.09/	n/a
D4	MVs/Brighton.GBR/44.11	Cell culture	MVs/Manchester.GBR/10.09/	n/a
D4	MVs/Brighton.GBR/49.11	Cell culture	MVs/Manchester.GBR/10.09/	KT732227
D4	MVs/Brighton.GBR/12.12/7	Oral fluid	MVs/Manchester.GBR/10.09/	KT732228
D4	MVs/London.GBR/20.12/	Oral fluid	MVs/Manchester.GBR/10.09/	KT732229
D4	MVs/London.GBR/20.12/3	Oral fluid	MVs/Manchester.GBR/10.09/	n/a
D4	MVs/Tonbridge.GBR/26.12/	Oral fluid	MVs/Manchester.GBR/10.09/	n/a
D8	MVi/Benni Mellal.MOR/05.03/	Cell culture	n/d	n/a
D8	MVs/Llandudno.GBR/6.12/	Oral fluid	MVi/Villupuram.IND/03.07/	n/a
D8	MVs/Llandudno.GBR/7.12/2	Oral fluid	MVi/Villupuram.IND/03.07/	KT732230
D8	MVs/Llandudno.GBR/8.12/	Oral fluid	MVi/Villupuram.IND/03.07/	n/a
D8	MVs/Birmingham.GBR/17.12/	Oral fluid	n/d	n/a
D8	MVs/London.GBR/18.12/10	Oral fluid	n/d	n/a
D8	MVs/London.GBR/20.12/6	Oral fluid	n/d	n/a
D8	MVs/London.GBR/22.12/3	Oral fluid	n/d	KT732231
D8	MVs/Taunton.GBR/27.12/3	Oral fluid	MVs/Taunton.GBR/27.12/	n/a
D8	MVs/London.GBR/28.12/	Oral fluid	MVs/Frankfurt_Main.DEU/17.11/	KT732232
D8	MVs/Sheffield.GBR/32.12/	Oral fluid	MVs/Taunton.GBR/27.12/	KT732233
D8	MVs/Sheffield.GBR/32.12/3	Oral fluid	MVs/Taunton.GBR/27.12/	KT732234
D8	MVs/Coventry.GBR/40.12/	Oral fluid	MVs/Taunton.GBR/27.12/	KT732235
D8	MVs/Coventry.GBR/42.12/2	Oral fluid	MVs/Taunton.GBR/27.12/	KT732236
D8	MVs/Gloucester.GBR/46.12/	Oral fluid	MVs/Taunton.GBR/27.12/	KT732237
D8	MVs/Gloucester.GBR/46.12/2	Oral fluid	MVs/Taunton.GBR/27.12/	KT732238
D8	MVs/Crewe.GBR/46.12/	Oral fluid	MVs/Taunton.GBR/27.12/	KT732239
D8	MVs/Hull.GBR/47.12	Cell culture	MVs/Taunton.GBR/27.12/	KT732240
D8	MVs/Derby.GBR/47.12/	Oral fluid	MVs/Taunton.GBR/27.12/	KT732241
D8	MVs/Darlington.GBR/48.12/	Oral fluid	MVs/Taunton.GBR/27.12/	n/a
D8	MVs/Darlington.GBR/49.12	Oral fluid	MVs/Taunton.GBR/27.12/	n/a
D8	MVs/Exeter.GBR/52.12/	Oral fluid	MVs/Taunton.GBR/27.12/	KT732242
D8	MVs/Swansea.GBR/4.13/2	Oral fluid	MVs/Swansea.GBR/4.13/	n/a
D8	MVs/Teeside.GBR/8.13/	Oral fluid	MVs/Taunton.GBR/27.12/	n/a
D8	MVs/Swansea.GBR/9.13/	Oral fluid	MVs/Swansea.GBR/4.13/	KT732245
D8	MVs/Teeside.GBR/9.13/3	Oral fluid	MVs/Taunton.GBR/27.12/	KT732244

D8	MVs/Teeside.GBR/9.13/4	Oral fluid	MVs/Taunton.GBR/27.12/	KT732246
D8	MVs/Teeside.GBR/9.13/5	Oral fluid	MVs/Taunton.GBR/27.12/	KT732243
D8	MVs/Swansea.GBR/10.13/	Oral fluid	MVs/Swansea.GBR/4.13/	KT732247
D8	MVs/Swansea.GBR/12.13/	Oral fluid	MVs/Swansea.GBR/4.13/	n/a
D8	MVs/Swansea.GBR/13.13/4	Oral fluid	MVs/Swansea.GBR/4.13/	KT732248
D8	MVs/Swansea.GBR/14.13/3	Cell culture	MVs/Frankfurt_Main.DEU/17.11/	n/a
D8	MVs/Swansea.GBR/14.13/4	Oral fluid	MVs/Swansea.GBR/4.13/	KT732250
D8	MVs/Lincoln.GBR/15.13/	Oral fluid	MVs/Taunton.GBR/27.12/	KT732256
D8	MVs/Swansea.GBR/15.13/	Oral fluid	MVs/Swansea.GBR/4.13/	KT732249
D8	MVs/Taunton.GBR/15.13/	Oral fluid	MVs/Swansea.GBR/4.13/	KT732252
D8	MVs/Ipswich.GBR/16.13/	Oral fluid	MVs/Taunton.GBR/27.12/	n/a
D8	MVs/Newcastle upon Tyne.GBR/16.13/	Oral fluid	MVs/Taunton.GBR/27.12/	KT732254
D8	MVs/Shrewsbury.GBR/16.13/	Oral fluid	MVs/Frankfurt_Main.DEU/17.11/	n/a
D8	MVs/Swansea.GBR/16.13/2	Oral fluid	MVs/Swansea.GBR/4.13/	KT732251
D8	MVs/Swansea.GBR/16.13/5	Oral fluid	MVs/Swansea.GBR/4.13/	KT732255
D8	MVs/Swansea.GBR/16.13/6	Oral fluid	MVs/Swansea.GBR/4.13/	n/a
D8	MVs/Teeside.GBR/16.13/6	Oral fluid	MVs/Taunton.GBR/27.12/	KT732253
D8	MVs/Teeside.GBR/16.13/8	Oral fluid	MVs/Taunton.GBR/27.12/	n/a
D8	MVs/Shrewsbury.GBR/17.13/	Oral fluid	MVs/Frankfurt_Main.DEU/17.11/	n/a
D8	MVs/Swansea.GBR/17.13/9	Oral fluid	MVs/Swansea.GBR/4.13/	KT732257
D8	MVs/Teeside.GBR/18.13/2	Oral fluid	MVs/Taunton.GBR/27.12/	KT732258
D8	MVs/London.GBR/19.13/	Oral fluid	MVs/Taunton.GBR/27.12/	n/a
D8	MVs/London.GBR/20.13/2	Cell culture	MVs/Taunton.GBR/27.12/	KT732260
D8	MVs/London.GBR/21.13/	Oral fluid	MVs/Taunton.GBR/27.12/	KT732259
D8	MVs/Manchester.GBR/30.13/	Cell culture	MVs/Taunton.GBR/27.12/	KT732261

n/d: no WHO named strain has been defined for this N-450 sequence.
n/a: the WGS-t was not obtained for this strain.
