# Supplementary material for: Assessment of the Utility of Whole Genome Sequencing of Measles Virus in the Characterisation of Outbreaks
Source: PLoS One. 2015 Nov 16;10(11):e0143081. doi: 10.1371/journal.pone.0143081 (PMC4646484; doi:10.1371/journal.pone.0143081)
Supplement: S2 Table — (PDF) [file pone.0143081.s005.pdf]

**S2 Table. Primers used for PCR enrichment.**

| Name | Sequence (5' > 3') |
|---|---|
| MeV-L1-3F | TTGGGTAAGGATAGATCAATCAATG |
| MeV-L1-3R | CTTGTTCTGAATTGAGTTCTCCAGG |
| MeV-L2-1F | GACATTGATACATATATCGTAGAGG |
| MeV-L2-1R | TTGACATGGCGTGCCTGC |
| MeV-L3-1F | TCTAGACTAGGTGCRAGRGGCCG |
| MeV-L3-1R | AGATTGTGGTACCAGATTCGGGTG |
| MeV-L4-1F | GAGACACCCATTAAAAAGGGC |
| MeV-L4-1R | GTCGAAGTCGTAGATCTCTG |
| MeV-L5-1F | CCTGATGACTCTCCTTGATG |
| MeV-L5-1R | CATCAGTGGGTAACATAAGGTC |
| MeV-L6-2F | CGGGAACTTCAGGAGAAAG |
| MeV-L6-3R | CCCCCCGTCTTGGAYTGTCG |
| MeV-L7nF[1] | AGACCACCAACCGCATCCC |
| MeV-L7nR[1] | GGCTCGCTCTCAGATTGTCG |
| MeV-L7-1F[2] | CGCACAAGCGACCGAGGTG |
| MeV-L7-3R[2] | CTAGGGCCGCACCTGCCAG |
| MeV-L8-1F | TGACTCGTTCCAGCCATCAATC |
| MeV-L8-1R | CACAATTGGCTATTAGGTTCCC |
| MeV-L9-1F | TTCATGCCAGAGGGGACTGTG |
| MeV-L9-1R | CGATCAAGCTCAGAAACATGACG |
| MeV-L10-1F | CAACGAGACCGGATAAATGC |
| MeV-L10-1R | CATGTCGGTTGGGGATTTC |
| MeV-L11-1F | GAGTGTTTGARGTAGGTGTTATCAG |
| MeV-L11-1R | AGCACACAGAAGTGACGGCAC |
| MeV-L12-1F | TGGCAACCTACGATACYTCCAG |
| MeV-L12-1R | ACAAGGTCACGAGAGATTAGC |
| MeV-L13-1F | AACTCACGGCTTGGCCTAG |
| MeV-L13-1R | CTTGGGAGGGTCGTAACGC |
| MeV-L14-1F | GCACGGAGGCAGTTGGCC |
| MeV-L14-1R | GGAATGGTGCTGATGGTCC |
| MeV-L15-1F | TGCCTTAATTGGAGATATGAGACC |
| MeV-L15-1R | GCAGTTATGTTCTTGAGGAGTC |
| MeV-L16-2F | CAGAAACATCGGTGATCCAGTAAC |
| MeV-L16-1R | GGAGTGATCACCCTTAGCTC |
| MeV-L17-1F | TGAGAGAACAGACATGAAGCTTG |
| MeV-L17-1R | CATTGACAAGCACCTTAAACACTC |
| MeV-L18-1F | AAGGACCATATGAATGAAATTTCAG |
| MeV-L18-4R | AAGAACAAGCCGTCTTCCC |
| MeV-L19-1F | CATGCTTTCCGCAGAATCGGG |
| MeV-L19-1R | TTGAATCTTGCCAACTCCCTGTAG |
| MeV-L20-1F | AGGTGATATCAACCCTACTCTG |
| MeV-L20-1R | CCAGACAAAGCTGGGAATAG |

[1] first PCR round

[2] second PCR round
